# Congenital Co-infections Among HIV-Exposed Infants Born to Mothers on Antiretroviral Treatment in the United States

**DOI:** 10.3389/fped.2022.894627

**Published:** 2022-06-16

**Authors:** Christiana Smith, Lori Silveira, Megan Crotteau, Krystle Garth, Jennifer Canniff, Kirk B. Fetters, Sarah Lazarus, Shannon Capraro, Adriana Weinberg, the CHIP Perinatal Team

**Affiliations:** ^1^Department of Pediatrics, University of Colorado, Aurora, CO, United States; ^2^Department of Medicine, Harbor-UCLA Medical Center, Torrance, CA, United States; ^3^University of Wisconsin, Madison, WI, United States; ^4^Department of Pediatrics, Children's Mercy Hospital, Kansas City, MO, United States; ^5^Departments of Medicine and Pathology, University of Colorado, Aurora, CO, United States

**Keywords:** HIV, cytomegalovirus, toxoplasmosis, hepatitis C, pregnancy, congenital, perinatal

## Abstract

**Background:**

Many women living with HIV (WLHIV) are co-infected with cytomegalovirus (CMV), *Toxoplasma gondii (T gondii*), and/or hepatitis C virus (HCV). The rates of congenital or perinatal transmission of these co-infections are not well defined in the current era, when most WLHIV receive antiretroviral therapy (ART) during pregnancy.

**Methods:**

Retrospective review of infants of WLHIV born between 2009–2019. Mothers were screened for antibodies to CMV, *T. gondii*, and HCV; chronic HCV infection was confirmed using plasma RNA PCR. Infants whose mothers had positive/unknown serostatus were screened for CMV using urine or saliva DNA PCR or culture at ≤3 weeks of life; *T. gondii* using serology at ≤1 month; and HCV using plasma RNA PCR at ≤6 months and serology at ≥12 months.

**Results:**

The study included 264 infants from 255 pregnancies in 191 mothers. At delivery, the median (IQR) CD4 count was 569 (406–748) cells/mm^3^ and plasma HIV load was 0 (0–24) RNA copies/mL. Among 243 infants born to CMV-seropositive (209) or CMV-missed serostatus (25) mothers, 163 (67.1%) were tested for CMV. Four infants had CMV detected, resulting in a rate of congenital infection of 2.5%. Among 65 infants from 54 (21.2%) pregnancies in *T. gondii*-seropositive women and 8 in women with unknown *T. gondii*-serostatus, one acquired congenital toxoplasmosis in the setting of acute maternal *T. gondii* infection. There were no episodes of vertical transmission from mothers with latent toxoplasmosis. Among 18 infants from 13 (5.1%) pregnancies in HCV RNA PCR-positive women and 4 in women with unknown HCV serostatus, there were no congenital or perinatal HCV transmissions.

**Conclusions:**

In a US cohort of pregnant WLHIV on ART, we identified high maternal CMV seroprevalence and a high rate of congenital CMV infection. We did not identify any congenital or perinatal transmissions of *T. gondii* or HCV among mothers who had latent or chronic infections. Our data support screening pregnant WLHIV and their infants for CMV and suggest that the rates of congenital and perinatal *T. gondii* and HCV infections among infants born to WLHIV on ART may be lower in the era of effective ART.

## Introduction

Many pregnant women living with HIV (WLHIV) are co-infected with other agents that establish chronic or latent infection, such as cytomegalovirus (CMV), *Toxoplasma gondii* (*T. gondii*), and/or hepatitis C virus (HCV). These infections may be more commonly transmitted to the fetus or neonate in the context of maternal HIV coinfection. The rates of congenital and/or perinatal transmission of these co-infections are not well defined in the current era, when few WLHIV are immune suppressed as a result of widespread routine administration of antiretroviral therapy (ART). Because there are no widely accepted interventions to prevent congenital and/or perinatal transmission from women with these chronic or latent infections, screening may not be routinely performed during pregnancy.

Congenital CMV infection occurs either during acute/primary maternal infection or reactivation of prior maternal infection. Reactivation is responsible for the majority of congenital CMV infections and occurs in both immune-competent and immunosuppressed mothers (1). Transmission rates among seropositive WLHIV have been reported as high as 2–6%, although many of these studies were performed before maternal ART was standard of care, and many infants acquired HIV, which may have impacted CMV transmission (9–15). Transmission of *T. gondii* to a fetus can occur either as a result of acute infection during pregnancy or reactivation of latent toxoplasmosis, which is rare and occurs only in immunocompromised women (16). While maternal immune status correlates inversely with risk of transmission, cases of congenital toxoplasmosis have been described in infants of WLHIV with CD4 counts >200 and even >500 cells/mm^3^; however, some reports do not specify whether transmission resulted from acute or chronic maternal infection (17). In pregnant women with detectable plasma HCV RNA, transmission to the infant can occur during pregnancy (~25% of transmissions) or delivery (~75% of transmissions) (20). In the US, routine testing of pregnant women for HCV is now recommended, but it is unclear what proportion of pregnant WLHIV are co-infected with HCV. In sum, there is a paucity of recent data on the seroprevalence of these co-infections and on congenital and perinatal transmission rates among WLHIV treated with ART in the US.

When infants acquire these congenital or perinatal infections, there can be potentially devastating consequences. Congenital CMV and toxoplasmosis can result in severe ophthalmologic and neurologic manifestations; congenital CMV is the leading non-genetic cause of sensorineural hearing loss in the United States (US) (1). Most infants with congenital CMV are asymptomatic at birth, but importantly, up to 15% of asymptomatic infants develop hearing loss, and late onset and progressive hearing loss can occur later in childhood (29, 30). Similarly, most infants who acquire congenital or perinatal HCV are asymptomatic at birth, but up to 80% develop chronic infection and hepatitis (24). Because of the severe potential outcomes associated with these infections, it is important to understand the epidemiology of mother-to-child transmission and establish guidelines for screening pregnant WLHIV and their infants. In this study, we sought to describe the epidemiology of CMV, *T. gondii*, and HCV maternal co-infections and rates of congenital and perinatal transmissions in a contemporary cohort of pregnant WLHIV treated with ART and residing in the US.

## Materials and Methods

### Study Design and Setting

In this retrospective cohort study, electronic health records were abstracted (EHR, Epic systems, Verona, WI) for all pregnant WLHIV followed by the Children's Hospital Immunodeficiency Program (CHIP) clinic between 1 August 2009 and 1 December 2019. The CHIP clinic is housed within Children's Hospital Colorado, a large tertiary care referral center in Aurora, Colorado. CHIP manages the majority of pregnant WLHIV in a 7-state region and follows HIV-exposed infants for the first 12–18 months of life. This study was approved by the Colorado Multiple Institutional Review Board (COMIRB) with a waiver of informed consent.

### Data Collection and Management

Demographic and clinical data were abstracted from the EHR and entered into standardized data collection forms developed in REDCap, hosted by the University of Colorado, Denver (31). EHR data included all available primary care, obstetric and HIV specialty care encounters, inpatient notes and diagnostic test results.

### Study Population

All women received ART during pregnancy and were counseled not to breastfeed. Infants received postnatal prophylaxis that consisted of a minimum of 4 weeks of oral zidovudine; infants at higher risk of HIV transmission received additional prophylactic antiretroviral drugs for up to 6 weeks. All pregnancies that resulted in a live birth during the study period were included.

### Laboratory Testing

Viral load and CD4 testing was performed monthly during pregnancy for WLHIV. “Entry” viral load and CD4 results were defined as the first results available after conception and before delivery. “Delivery” viral load and CD4 results were defined as the results available closest to the date of infant birth, within the range of 45 days prior to infant birth to 100 days after infant birth. Infants were tested for HIV using RNA or DNA PCR at birth, 2 weeks of age, 4–6 weeks of age, and 4–6 months of age. HIV antigen/antibody test was performed at 12–24 months of age to document sero-reversion.

Mothers were screened for co-infections using serologic testing for CMV, *T. gondii*, and HCV at least once during pregnancy, typically in the first or second trimester. Repeat CMV screening was performed later in pregnancy for mothers with a clinical illness that could be compatible with acute CMV infection. Screening for *T. gondii* was not typically repeated in the third trimester, as *T. gondii* is not endemic to the dry climate and high elevation of the US mountain west; therefore, mothers were at low risk for primary infection during pregnancy. In mothers seropositive for HCV, active HCV infection was confirmed using RNA PCR. Screening was repeated during each pregnancy except for mothers known to be seropositive from prior pregnancies.

Infants of seropositive mothers or mothers with unknown serostatus were screened for CMV using urine or saliva DNA PCR or culture in the first 3 weeks of life, *T. gondii* using serologic testing in the first month of life (with repeat testing at 4 months and 12 months if initial IgG detectable), and HCV using RNA PCR by 6 months of life and serologic testing after 12 months of life.

### Statistical Analysis

Demographics are reported using descriptive statistics; medians (interquartile ranges) for continuous variables or counts (percent) for categorical variables. When calculating rates of transmission, each mother-infant dyad was considered an independent transmission opportunity. Ninety-five percent confidence intervals for the binomial distribution were calculated using the Clopper-Pearson exact method.

## Results

During the study period, 191 mothers had 255 pregnancies, 9 of which were twin gestations, for a total of 264 live-born infants. The demographics of the WLHIV and their infants are described in Table 1. Most pregnancies (66.3%) occurred in women who identified as non-white race and nearly half of pregnancies (44.3%) occurred in women who were internationally-born. Mothers generally had well-controlled HIV; at delivery the median (IQR) CD4 count was 569 (406–748) cells/mm^3^ and viral load was 0 (0–24) copies/mL. Only 8.2% of pregnancies occurred in women who had ever met CDC criteria for Stage 3 HIV infection (AIDS). Nearly all (92.2%) pregnancies were treated with ART for at least 12 weeks prior to delivery. Deliveries occurred at a median of 38.4 (37.6–39.4) weeks gestational age; 48 (18%) infants were born preterm.

**Table 1:** Demographics of mothers^a^ and infants during the study period.

**Pregnancy characteristics** **(*N* = 255)**	**Number evaluable**	**Median (IQR) or count (%)**
Maternal birthplace	247	
United States		134 (52.5%)
Africa		78 (30.6%)
Latin America		22 (8.6%)
East and Southeast Asia		11 (4.3%)
Eastern Europe		2 (0.8%)
Maternal race	211	
Black non-Hispanic		102 (40.0%)
Hispanic		54 (21.2%)
White non-Hispanic		42 (16.5%)
Other		13 (5.1%)
Toxicology screen positive during current pregnancy	255	23 (9.0%)
Maternal age at delivery (years)	255	30 (26–34)
Gravidity	252	2 (1–4)
Parity	253	1 (1–2)
CD4 count at entry^b^ (cells/mm^3^)	244	555 (387–780)
CD4 count at delivery^c^ (cells/mm^3^)	173	569 (406–748)
Viral load at entry^b^ (copies/mL)	248	25 (0–6105)
Viral load at delivery^c^ (copies/mL)	253	0 (0–24)
AIDS diagnosis ever	243	21 (8.2%)
Duration of any ART during pregnancy (weeks)	255	36.4 (23–39)
ART received for at least 12 weeks before delivery	255	235 (92.2%)
Adherence self-report	222	
100% of doses		184 (72.2%)
75–100% of doses		26 (10.2%)
50–75% of doses		6 (2.4%)
<50% of doses		6 (2.4%)
Mechanism of delivery	251	
Vaginal		119 (46.7%)
Assisted vaginal		3 (1.2%)
Cesarean		129 (50.6%)
Twin gestation	255	9 (3.5%)
Infant characteristics (*N* = 264)	Number evaluable	Median (IQR) or count (%)
Gestational age at delivery (weeks)	262	38.4 (37.6–39.4)
Infants born <37 weeks gestation	262	48 (18.3%)
Infant birthweight (grams)	260	3,014 (2,668–3,320)
Infant sex, male	264	135 (51.1%)

a *Some mothers are included more than once in the table; 191 women had 255 pregnancies. Including 9 twins, there were 264 live-born infants during the study period*.

b *Entry viral load and CD4 results were defined as the first results available after conception and before delivery*.

c *Delivery viral load and CD4 results were defined as the results available closest to the date of infant birth, within the range of 45 days prior to infant birth to 100 days after infant birth*.

*AIDS, acquired immunodeficiency syndrome; ART, antiretroviral treatment; IQR, interquartile range*.

### HIV Transmissions

One infant in this cohort acquired HIV in utero (0.37%). The infant's mother immigrated to the US from an African country late in her pregnancy. Her first prenatal visit in the US occurred at 38 weeks gestational age, when HIV infection was diagnosed. The CD4 count was 445 cells/mm^3^ and viral load was 47,000 copies/mL. The mother immediately initiated ART and a scheduled cesarean section was performed the next day. However, the infant had two detectable HIV DNA/RNA PCRs performed on days of life 0 and 2, which established the diagnosis of in utero HIV infection. All other infants in this cohort were determined to be HIV-uninfected.

### Cytomegalovirus Seroprevalence and Transmissions

CMV-seropositive status was documented in 209 (82.0%) pregnancies among WLHIV. CMV-seronegative status was documented in 21 (8.2%) pregnancies, and missing status in 25 (9.8%) pregnancies. The majority of tests (82.9%) were performed in the first or second trimesters. Three seropositive mothers had detectable anti-CMV IgM antibodies during pregnancy. One of these mothers, who was tested in the second trimester, had a qualitatively positive, but not quantifiable whole blood CMV DNA PCR. The other two mothers, who were tested in the first and third trimesters, respectively, had high anti-CMV IgG avidity, indicating that the primary infection had occurred ≥4 months before the diagnosis; one of these mothers had an undetectable CMV DNA PCR and the other mother did not have CMV DNA PCR performed. None of the mothers with detectable anti-CMV IgM antibodies transmitted CMV to their infants.

Among 243 infants whose mothers were CMV-seropositive or unknown, 163 (67.1%) infants had CMV test results available. Of those, 4 infants had a detectable DNA PCR in the first 3 weeks of life, resulting in a prevalence of congenital CMV of 2.5% (95% CI: 0.7–6.2%). The rate of congenital CMV transmission was 1.6% if all of the infants with missing CMV test results were assumed to have negative testing, and 2.6% if excluding all mothers and infants with missing CMV test results.

Fourteen CMV-screened infants were the products of twin gestation, of whom one infant had detectable CMV DNA in urine. Both infants in the twin pair with discordant results were tested for CMV twice in the first 3 weeks of life; one infant was repeatedly positive and the other infant was repeatedly negative.

Of the 4 infants with congenital CMV, all mothers received ART during pregnancy. Two mothers received ART continuously from prior to conception until after delivery. Both of these mothers had undetectable viral loads throughout pregnancy except for 1 “blip” in each mother to <100 copies/mL and subsequent viral suppression. A third mother initiated ART in the 2^nd^ trimester of pregnancy and her viral load quickly suppressed and remained undetectable thereafter; her CD4 count was 64 cells/mm^3^ early in pregnancy and 197 cells/mm^3^ at delivery. The fourth mother was difficult to engage in prenatal care due to substance dependence and incarceration. She initiated ART 2 weeks prior to delivery. At delivery, her viral load was 1,000 copies/mL and her CD4 count was 180 cells/mm^3^. All 4 infants were asymptomatic at birth. One infant failed a newborn hearing screen, but subsequent hearing evaluations were normal. This infant, who was exposed to substances in utero and born at 36 weeks gestational age, was diagnosed with developmental delay that required physical and occupational therapy. Another infant, whose newborn hearing screen was normal, developed mild hearing impairment later in life. None of these 4 infants were treated with antivirals directed at CMV infection.

Among the 142 pregnancies in CMV-seropositive mothers that did not result in congenital CMV transmission, the median (IQR) CD4 count at delivery was 573 (437–738) cells/mm^3^ and viral load was 0 (0–15) copies/mL. The median duration of ART during those pregnancies was 37 weeks. There were 7 mothers treated with fewer than 12 weeks of ART prior to delivery. One of these mothers was the mother whose infant acquired HIV, who received only 1 day of ART prior to delivery. The remaining six women were either diagnosed with HIV late in pregnancy (*n* = 4) or had previously established HIV infection but were difficult to engage in care (*n* = 2). All six pregnancies were treated with ART (range of 2 to 11 weeks), and the HIV viral loads at delivery ranged from 0 to 2,600.

### Toxoplasma Gondii Seroprevalence and Transmissions

*T. gondii*-seropositive status was documented in 54 (21.2%) pregnancies among WLHIV. *T. gondii*-seronegative status was documented in 193 (75.7%) pregnancies and missing status in 8 (3.1%) pregnancies. The majority of tests (77.4%) were performed in the first or second trimesters. One seropositive mother, described below, had evidence of acute *T. gondii* infection during pregnancy.

Among 65 infants whose mothers were *T. gondii*-seropositive or unknown, 50 (76.9%) had *T. gondii* test results available. Of those, congenital toxoplasmosis was diagnosed in one infant who was born to the mother with acute *T. gondii* infection. The remaining 49 infants had negative serologic testing; there were no known cases of reactivation of latent toxoplasmosis with transmission to the fetus. Assuming binomial variation, the upper confidence limit of the estimate of 0 *T. gondii* transmissions is 5.5%.

The infant who acquired congenital toxoplasmosis is the same infant who acquired HIV in utero, described above. This infant's mother had a *T. gondii* serologic panel sent during her hospitalization for cesarean delivery. The results became available after the mother and infant were discharged home, and demonstrated detectable maternal anti-*T. gondii* IgM, IgA, and IgG, indicating recent infection. The infant was subsequently tested, and also had detectable anti-*T. gondii* IgM, IgA, and IgG. The infant's workup was notable for intracranial calcifications and chorioretinitis. Hearing evaluation was normal. The infant initiated treatment with pyrimethamine, sulfadiazine, and leucovorin on day of life 20. Medications directed against *T. gondii* were continued for the first year of life, with the exception that pyrimethamine was held several times and the sulfadiazine was briefly replaced with clindamycin around 3–4 months of age, due to drug-induced neutropenia. The infant's growth, development, and neurologic exam were normal at age 3 years, when the family relocated outside of the CHIP referral region.

### Hepatitis C Seroprevalence and Transmissions

HCV-seropositive status was documented in 16 (6.3%) pregnancies among WLHIV. HCV-seronegative status was documented in 235 (92.2%) pregnancies, and missing status in 4 (1.6%) pregnancies. The majority of tests (80.0%) were performed in the first or second trimesters. Among the HCV-seropositive mothers, 13 pregnancies occurred in women with detectable RNA PCR, resulting in a prevalence of chronic active HCV infection of 5.1% in this cohort.

There were no known congenital or perinatal transmissions of HCV. Among 18 infants whose mothers had detectable HCV RNA PCR or had unknown HCV status, 14 (77.8%) had HCV test results available, all of whom had negative results. Assuming binomial variation, the upper confidence limit of the estimate of 0 HCV transmissions is 18.5%.

## Discussion

In this US cohort of pregnant WLHIV treated with ART, the majority of whom had well-controlled HIV, we identified high maternal CMV seroprevalence (82%) and a high rate of congenital CMV infection (2.5%). CMV seroprevalence among women of childbearing age varies from 50% in high-income countries to >95% in low and middle-income countries (2, 3). Within high-income countries, seroprevalence correlates with nonwhite race, internationally-born status, and lower socioeconomic backgrounds; some studies have also shown higher seroprevalence among people living with HIV than in the general population (3–5). In areas with near-universal maternal CMV seroprevalence, the incidence of congenital CMV is 6–8 per 1,000 infants, resulting in a mother-to-child transmission rate of <1% (6–8). In our cohort of WLHIV, congenital CMV infection occurred at a more than 2-fold higher rate than is observed in the general population. Of note, approximately one-third of infants at risk for congenital CMV did not have test results available. Even if all of the infants whose test results were missing had negative testing for CMV, we would have identified 4 transmissions among 243 infants at risk, which would have resulted in a rate of congenital CMV of 1.6%, still higher than is observed in the general population. It is important to note that the lower limit of our 95%CI for CMV transmissions was 0.7%, which is similar to the rate of 0.6–0.8% described in highly CMV-seropositive, HIV-uninfected populations (6–8). In addition, two infants with congenital CMV were born to women who initiated ART late in pregnancy; therefore, it is possible that transmission rates may be lower among WLHIV who are virally suppressed on ART prior to conception. Still, our results are in agreement with prior reports that WLHIV are more likely to reactivate and transmit CMV to their infants than women without HIV (9–15).

Among the 4 cases of congenital CMV in our cohort, only one developed sequelae attributed to CMV infection. This infant developed mild hearing loss after the newborn period, which did not require any intervention. Another infant had developmental delay requiring therapies, but was substance-exposed in utero and born late-preterm, which precluded the attribution of defects to a direct effect of CMV. All four CMV transmissions in our cohort occurred as a result of reactivation of latent maternal CMV infection. We identified three mothers with detectable anti-CMV IgM antibodies concerning for acute CMV infection during pregnancy, but two of these mothers had high anti-CMV IgG avidity, which decreases the likelihood that their CMV infection was acute.

We did not identify any congenital or perinatal transmissions of *T. gondii* or HCV among HIV-exposed infants whose mothers were virologically suppressed on ART. The one congenital toxoplasmosis case in our cohort occurred in the setting of acute maternal *T. gondii* infection in a mother with uncontrolled HIV replication. This infant acquired both congenital toxoplasmosis and in utero HIV infection; these two infections have been previously described to occur simultaneously and having one in utero infection may increase the risk of acquiring the other (32). If this mother had presented to care earlier in pregnancy, her HIV and toxoplasmosis could have been treated, and transmission of these infections to the infant could potentially have been avoided.

Importantly, acute *T. gondii* infection during pregnancy is a high risk scenario for fetal infection, and transmission in this setting occurs in both immune-competent and immunosuppressed mothers. In contrast, reactivation of latent toxoplasmosis and transmission to the fetus is rare, but has been reported in WLHIV. Congenital transmission of toxoplasmosis from a seropositive WLHIV is estimated to occur at a rate of <4% (17–19). While we identified zero transmissions among the 65 infants at risk in our cohort, the upper confidence limit of this estimate was 5.5%, which is consistent with the previously reported rate. While our cohort was small, and only 21.2% of mothers were seropositive for *T. gondii*, our results provide reassurance that congenital toxoplasmosis may occur less frequently among WLHIV who are virologically suppressed on ART than among WLHIV on less optimal treatment.

Similarly, we identified zero instances of congenital or perinatal HCV infection among the 18 infants at risk in our cohort, which suggests that maternal immune health may be protective against transmission of HCV to the infants of WLHIV. Mother-to-child HCV transmission has been estimated to occur at a rate of 5–6% among women without HIV and 10–11% among WLHIV; however, many studies in WLHIV were performed before maternal ART was standard of care, and many infants were co-infected with HIV (25–28). Importantly, the upper confidence limit of our estimate was 18.5%, which is similar to the previously reported rate of 10–11% among WLHIV (25). The small number of women with chronic active HCV infection in our cohort may have limited our ability to estimate congenital or perinatal transmission rates.

Notably, the prevalence of 5% chronic HCV infections among WLHIV in our cohort is much higher than the prevalence reported for women of childbearing age in the US in general (0.5–1%), and is similar to the prevalence identified in WLHIV in another study from New York State (3.8%) (21–24). It is important to obtain a precise estimate of the risk of HCV vertical transmission in the context of maternal HIV co-infection. Treatment of HCV infection during pregnancy is currently under investigation and may become available in the next few years. However, due to the small number of pregnancies complicated by chronic HCV infection, it will take many years to gather data on the risk of infant toxicity and congenital defects that may be associated with the use of anti-HCV medications in pregnancy. Moreover, infants born to women with HIV are already exposed to ART in utero, which carries its own risks of toxicity (33). Thus, the benefit of HCV treatment during pregnancy will need to be weighed against the risk of in utero drug exposure, especially among the population of WLHIV. Importantly, people with HIV should be screened for HCV and treated, if appropriate, as soon as the diagnosis of HIV is established (34). Thus, the decision to treat HCV during pregnancy in WLHIV only applies to those in whom the diagnoses of HIV and HCV are made after conception.

Our results support screening of pregnant WLHIV and their infants for CMV. The Department of Health and Human Services (HHS) recommends screening only HIV-exposed infants whose mothers had acute CMV infection during pregnancy and infants with in utero HIV infection, with an added consideration for screening all HIV-exposed infants because in utero HIV transmission is often not ruled out during the window for congenital CMV testing (in the first 3 weeks of life) (35). Our data suggest that HIV-exposed, uninfected infants are at increased risk of congenital CMV, so screening them may be warranted even if HIV infection has been ruled out. Infants who are identified as having congenital CMV should be followed closely and evaluated for hearing loss at 6 month intervals, even if they are asymptomatic and have a normal hearing evaluation at birth (35).

The HHS recommends routine screening of pregnant WLHIV for latent *T. gondii* infection and antimicrobial prophylaxis against toxoplasmosis in seropositive WLHIV whose CD4 counts are <100 cells/mm^3^ (35). Routine screening for HCV is indicated for all people living with HIV upon entry to care (34). In addition, screening of all pregnant women for HCV has been recommended in the US since 2019, and has been shown to be cost-effective when the prevalence of HCV is ≥0.03% (36, 37). While we are in agreement with screening for both of these infections in pregnant WLHIV, we note that the rates of congenital and perinatal *T. gondii* and HCV infections among infants born to WLHIV who are virologically suppressed on ART may be lower than previously described.

Limitations of this study include its retrospective nature and accordingly, the substantial proportion of mothers and infants who had missing data. We may have underestimated transmission rates for CMV by including infants of mothers with missing serologic screening results in the “at risk” group. The total number of pregnancies included in the study was relatively small, which limited our ability to identify congenital or perinatal infections and resulted in wide confidence intervals around our estimates of transmission, especially for *T. gondii* and HCV. In addition, this study was performed at a single site, which may limit the generalizability of our results. However, our cohort included a diverse group of mothers and infants that is likely to be representative of the demographics of pregnant WLHIV managed at other urban US hospital-based clinics.

In conclusion, we identified a high rate of congenital CMV among infants born to WLHIV treated with ART and residing in the US, and no congenital or perinatal transmissions of *T. gondii* or HCV among HIV-exposed infants whose mothers were on ART during pregnancy. Our data support screening efforts of pregnant WLHIV and their infants for CMV and underscore the need for additional studies on transmission of *T. gondii* and HCV in this population.

## Data Availability Statement

The raw data supporting the conclusions of this article will be made available by the authors, without undue reservation.

## Ethics Statement

The studies involving human participants were reviewed and approved by the Colorado Multiple Institutional Review Board (COMIRB). Written informed consent from the participants' legal guardian/next of kin was not required to participate in this study in accordance with the national legislation and the institutional requirements.

## Author Contributions

CS was responsible for study design, data collection, data interpretation, and authored the first draft. LS was responsible for data analysis, and critically reviewed and revised the manuscript. MC, KG, JC, KF, SL, and SC were responsible for data collection and critically reviewed and revised the manuscript. AW was responsible for study design, data interpretation, and critically reviewed and revised the manuscript. All authors contributed to the article and approved the submitted version.

## Funding

Institutional funding supported this study. The CHIP clinic receives funding from award numbers: HHSN275201800001I; 5P01HD103133-02; 5U01HD068063-05; 5U01HD068040474-15S1; UM1 AI068619; ENVHL-202158734-00; X0700056; H12HA24784. Through access to University of Colorado REDCap, this project was supported by NIH/NCATS Colorado CTSA Grant Number UL1 TR002535. Its contents are the authors' sole responsibility and do not necessarily represent official NIH views.

## Conflict of Interest

AW receives research grants from Merck, GlaxoSmithKline and Janssen (moneys to University of Colorado) and personal fees from Merck, Seqirus and Curevo. The remaining authors declare that the research was conducted in the absence of any commercial or financial relationships that could be construed as a potential conflict of interest.

## Publisher's Note

All claims expressed in this article are solely those of the authors and do not necessarily represent those of their affiliated organizations, or those of the publisher, the editors and the reviewers. Any product that may be evaluated in this article, or claim that may be made by its manufacturer, is not guaranteed or endorsed by the publisher.

## References

[1] American Academy of Pediatrics. Cytomegalovirus Infection. In: Kimberlin DW Barnett ED Lynfield R Sawyer MH editors. Red Book: 2021–2024 Report of the Committee on Infectious Diseases. Itasca, IL: American Academy of Pediatrics (2021).

[2] Kenneson A Cannon MJ. Review and meta-analysis of the epidemiology of congenital cytomegalovirus (CMV) infection. Rev Med Virol. (2007) 17:253–76. 10.1002/rmv.535 17579921

[3] Cannon MJ Schmid DS Hyde TB. Review of cytomegalovirus seroprevalence and demographic characteristics associated with infection. Rev Med Virol. (2010) 20:202–13. 10.1002/rmv.655 20564615

[4] N'Diaye DS Yazdanpanah Y Krivine A Andrieu T Rozenberg F Picone O. Predictive factors of cytomegalovirus seropositivity among pregnant women in Paris, France. PLoS ONE. (2014) 9:e89857. 10.1371/journal.pone.0089857 24587077 PMC3933677

[5] Hoehl S Berger A Ciesek S Rabenau HF. Thirty years of CMV seroprevalence-a longitudinal analysis in a German university hospital. Eur J Clin Microbiol Infect Dis. (2020) 39:1095–102. 10.1007/s10096-020-03814-x 31989374 PMC7225192

[6] Rico A Dollard SC Valencia D Corchuelo S Tong VT Laiton-Donato K. Epidemiology of cytomegalovirus Infection among mothers and infants in Colombia. J Med Virol. (2021) 93:6393–7. 10.1002/jmv.26815 33475162 PMC8292416

[7] Wang S Wang T Zhang W Liu X Wang X Wang H. Cohort study on maternal cytomegalovirus seroprevalence and prevalence and clinical manifestations of congenital infection in China. Medicine. (2017) 96:e6007. 10.1097/MD.0000000000006007 28151899 PMC5293462

[8] Yamamoto AY Anastasio ART Massuda ET Isaac ML Manfredi AKS Cavalcante JMS. Contribution of congenital cytomegalovirus infection to permanent hearing loss in a highly seropositive population: the Brazilian cytomegalovirus hearing and maternal secondary infection study. Clin Infect Dis. (2020) 70:1379–84. 10.1093/cid/ciz413 31102409 PMC7931844

[9] Doyle M Atkins JT Rivera-Matos IR. Congenital cytomegalovirus infection in infants infected with human immunodeficiency virus type 1. Pediatr Infect Dis J. (1996) 15:1102–6. 10.1097/00006454-199612000-00010 8970220

[10] Kovacs A Schluchter M Easley K Demmler G Shearer W La Russa P. Cytomegalovirus infection and HIV-1 disease progression in infants born to HIV-1-infected women. Pediatric Pulmonary and Cardiovascular Complications of Vertically Transmitted HIV Infection Study Group. N Engl J Med. (1999) 341:77–84. 10.1056/NEJM199907083410203 10395631 PMC4280563

[11] Guibert G Warszawski J Le Chenadec J Blanche S Benmebarek Y Mandelbrot L. Decreased risk of congenital cytomegalovirus infection in children born to HIV-1-infected mothers in the era of highly active antiretroviral therapy. Clin Infect Dis. (2009) 48:1516–25. 10.1086/598934 19388872

[12] Frederick T Homans J Spencer L Kramer F Stek A Operskalski E. The effect of prenatal highly active antiretroviral therapy on the transmission of congenital and perinatal/early postnatal cytomegalovirus among HIV-infected and HIV-exposed infants. Clin Infect Dis. (2012) 55:877–84. 10.1093/cid/cis535 22675157

[13] Duryea EL Sanchez PJ Sheffield JS Jackson GL Wendel GD McElwee BS. Maternal human immunodeficiency virus infection and congenital transmission of cytomegalovirus. Pediatr Infect Dis J. (2010) 29:915–8. 10.1097/INF.0b013e3181e0ce05 20431424

[14] Gantt S Leister E Jacobsen DL Boucoiran I Huang ML Jerome KR. Risk of congenital cytomegalovirus infection among HIV-exposed uninfected infants is not decreased by maternal nelfinavir use during pregnancy. J Med Virol. (2016) 88:1051–8. 10.1002/jmv.24420 26519647 PMC4818099

[15] Richardson BA John-Stewart G Atkinson C Nduati R Asbjornsdottir K Boeckh M. Vertical cytomegalovirus transmission from HIV-infected women randomized to formula-feed or breastfeed their infants. J Infect Dis. (2016) 213:992–8. 10.1093/infdis/jiv515 26518046 PMC4760415

[16] Maldonado YA Read JS Committee Committee On Infectious D. Diagnosis, treatment, and prevention of congenital toxoplasmosis in the United States. Pediatrics. (2017) 139:e20163860. 10.1542/peds.2016-3860 28138010

[17] Campos FA Andrade GM Lanna Ade P Lage BF Assumpcao MV Pinto JA. Incidence of congenital toxoplasmosis among infants born to HIV-coinfected mothers: case series and literature review. Braz J Infect Dis. (2014) 18:609–17. 10.1016/j.bjid.2014.05.008 25017666 PMC9425224

[18] Azevedo KM Setubal S Lopes VG Camacho LA Oliveira SA. Congenital toxoplasmosis transmitted by human immunodeficiency-virus infected women. Braz J Infect Dis. (2010) 14:186–9. 10.1016/S1413-8670(10)70036-2 20563448

[19] Low incidence of congenital toxoplasmosis in children born to women infected with human immunodeficiency virus. European collaborative study and research network on congenital toxoplasmosis. Eur J Obstet Gynecol Reprod Biol. (1996) 68:93–6. 10.1016/0301-2115(96)02497-9 8886688

[20] Terrault NA Levy MT Cheung KW Jourdain G. Viral hepatitis and pregnancy. Nat Rev Gastroenterol Hepatol. (2021) 18:117–30. 10.1038/s41575-020-00361-w 33046891

[21] Centers for Disease Control Prevention. Hepatitis C Questions and Answers for Health Professionals. Available online at: https://www.cdc.gov/hepatitis/hcv/hcvfaq.htm#section1 (accessed February 21, 2022).

[22] Ghazaryan L Smith L Parker M Flanigan C Pulver W Sullivan T. Hepatitis C seroprevalence among HIV-infected childbearing women in New York state in 2006. Matern Child Health J. (2016) 20:550–5. 10.1007/s10995-015-1853-4 26520159

[23] Panel on Treatment of HIV During Pregnancy and Prevention of Perinatal Transmission. Recommendations for the Use of Antiretroviral Drugs During Pregnancy and Interventions to Reduce Perinatal HIV Transmission in the United States.. Available online at: https://clinicalinfo.hiv.gov/en/guidelines/perinatal (accessed February 21, 2022).

[24] Hepatitis C Virus Infection. In: Kimberlin DW editors. Red Book: 2021–2024 Report of the Committee on Infectious Diseases. Itasca, IL: American Academy of Pediatrics (2021).

[25] Benova L Mohamoud YA Calvert C Abu-Raddad LJ. Vertical transmission of hepatitis C virus: systematic review and meta-analysis. Clin Infect Dis. (2014) 59:765–73. 10.1093/cid/ciu447 24928290 PMC4144266

[26] Thomas DL Villano SA Riester KA Hershow R Mofenson LM Landesman SH. Perinatal transmission of hepatitis C virus from human immunodeficiency virus type 1-infected mothers. Women and infants transmission study. J Infect Dis. (1998) 177:1480–8. 10.1086/515315 9607823

[27] Zanetti AR Tanzi E Romano L Principi N Zuin G Minola E. Multicenter trial on mother-to-infant transmission of GBV-C virus. The lombardy study group on vertical/perinatal hepatitis viruses transmission. J Med Virol. (1998) 54:107-12. 10.1002/(sici)1096-9071(199802)54:2<107::aid-jmv7>3.0.co;2-a 9496368

[28] Tovo PA Palomba E Ferraris G Principi N Ruga E Dallacasa P. Increased risk of maternal-infant hepatitis C virus transmission for women coinfected with human immunodeficiency virus type 1. Italian Study Group for HCV Infection in Children. Clin Infect Dis. (1997) 25:1121–4. 10.1086/516102 9402369

[29] Fowler KB McCollister FP Sabo DL Shoup AG Owen KE Woodruff JL. A targeted approach for congenital cytomegalovirus screening within newborn hearing screening. Pediatrics. (2017) 139:e20162128. 10.1542/peds.2016-2128 28049114 PMC5260148

[30] Dahle AJ Fowler KB Wright JD Boppana SB Britt WJ Pass RF. Longitudinal investigation of hearing disorders in children with congenital cytomegalovirus. J Am Acad Audiol. (2000) 11:283–90. 10.1055/s-0042-1748054 10821506

[31] Harris PA Taylor R Thielke R Payne J Gonzalez N Conde JG. Research electronic data capture (REDCap)–a metadata-driven methodology and workflow process for providing translational research informatics support. J Biomed Inform. (2009) 42:377–81. 10.1016/j.jbi.2008.08.010 18929686 PMC2700030

[32] Delicio AM Milanez H Amaral E Morais SS Lajos GJ. e Silva JL. Mother-to-child transmission of human immunodeficiency virus in aten years period. Reprod Health. (2011) 8:35. 10.1186/1742-4755-8-35 22129112 PMC3247874

[33] Van Dyke RB Chadwick EG Hazra R Williams PL Seage GR. 3rd. The PHACS SMARTT study: assessment of the safety of in utero exposure to antiretroviral drugs. Front Immunol. (2016) 7:199. 10.3389/fimmu.2016.00199 27242802 PMC4876360

[34] Panel on Guidelines for the Prevention Treatment of Opportunistic Infections in Adults Adolescents with HIV. Guidelines for the Prevention and Treatment of Opportunistic Infections in Adults and Adolescents With HIV: Recommendations From the Centers for Disease Control and Prevention, the National Institutes of Health, and the HIV Medicine Association of the Infectious Diseases Society of America. Available online at: https://clinicalinfo.hiv.gov/en/guidelines/adult-and-adolescent-opportunistic-infection (accessed February 21, 2022).

[35] Panel on Opportunistic Infections in HIV-Exposed HIV-Infected Children. Guidelines for the Prevention and Treatment of Opportunistic Infections in HIV-Exposed and HIV-Infected Children. Department of Health and Human Services. Available online at: https://clinicalinfo.hiv.gov/en/guidelines/pediatric-opportunistic-infection.

[36] American Association for the Study of Liver Diseases the Infectious Diseases Society of America. Hcv Guidance: Recommendations for Testing, Managing, and Treating Hepatitis C. (2020). Available online at: https://www.hcvguidelines.org/https://www.hcvguidelines.org/(accessed February 21, 2022). 10.1002/hep.31060 PMC9710295 31816111

[37] Chaillon A Rand EB Reau N Martin NK. Cost-effectiveness of universal hepatitis C virus screening of pregnant women in the United States. Clin Infect Dis. (2019) 69:1888–95. 10.1093/cid/ciz063 30689769 PMC7188080

